# Natural variation in virulence of the entomopathogenic fungus *Beauveria bassiana* against malaria mosquitoes

**DOI:** 10.1186/1475-2875-13-479

**Published:** 2014-12-06

**Authors:** Claudio A Valero-Jiménez, Alfons JM Debets, Jan AL van Kan, Sijmen E Schoustra, Willem Takken, Bas J Zwaan, Constantianus JM Koenraadt

**Affiliations:** Laboratory of Genetics, Wageningen University, P.O Box 309, 6700 AH Wageningen, The Netherlands; Laboratory of Entomology, Wageningen University, P.O. Box 8031, 6700 EH Wageningen, The Netherlands; Laboratory of Phytopathology, Wageningen University, P.O Box 8025, 6700 EE Wageningen, The Netherlands

**Keywords:** Entomopathogenic fungi, *Beauveria bassiana*, Vector control, Virulence

## Abstract

**Background:**

Insecticide resistance is greatly hampering current efforts to control malaria and therefore alternative methods are needed. Entomopathogenic fungi have been proposed as an alternative with a special focus on the cosmopolitan species *Beauveria bassiana*. However, few studies have analysed the effects of natural variation within fungal isolates on mosquito survival, and the implications and possible exploitation for malaria control.

**Methods:**

Laboratory bioassays were performed on adult female mosquitoes (*Anopheles coluzzii*) with spores from 29 isolates of *B. bassiana*, originating from different parts of the world. In addition, phenotypic characteristics of the fungal isolates such as sporulation, spore size and growth rate were studied to explore their relationship with virulence.

**Results:**

All tested isolates of *B. bassiana* killed *An. coluzzii* mosquitoes, and the rate at which this happened differed significantly among the isolates. The risk of mosquitoes dying was around ten times higher when they were exposed to the most virulent as compared to the least virulent isolate. There was significant variation among isolates in spore size, growth rate and sporulation, but none of these morphological characteristics were correlated, and thus predictive, for the ability of the fungal isolate to kill malaria mosquitoes.

**Conclusions:**

This study shows that there is a wide natural variation in virulence of isolates of *B. bassiana*, and that selecting an appropriate fungal isolate is highly relevant in killing and thus controlling malaria mosquitoes, particularly if used as part of an integrated vector management strategy. Also, the wide variation observed in virulence offers the opportunity to better understand the molecular and genetic mechanisms that drive this variation and thus to address the potential development of resistance against entomopathogenic fungi.

## Background

Although globally malaria mortality rates have fallen by 45% between 2000 and 2012, malaria is still killing an estimated 627,000 people each year [1]. An effective way to alleviate the burden of malaria is to control its vector (anopheline mosquitoes) using insecticides. This can be achieved either through the use of insecticide-treated bed nets (ITNs) or through indoor residual spraying of insecticides (IRS). However, because of rapidly expanding insecticide resistance, there is a need to find alternatives to control the mosquitoes [1]. Entomopathogenic fungi have been proposed as novel biological control agent to kill malaria mosquitoes [2–4]. Such fungi have already been used on a wide scale to control beetles [5], locusts [6] and other pest insects in agriculture.

Spores of hypocrealean entomopathogenic fungi are able to infect insects, including mosquitoes, via attachment to the insect’s epicuticle [7]. Spores will penetrate the insect’s cuticle by forming a germ tube and appressorium. The latter structure uses mechanical pressure and produces cuticle-degrading enzymes for further penetration [8, 9]. Once the fungal structures reach the haemocoel, they are able to use the insect nutrients and grow. If the fungi are able to overcome the host immune defences, the host will die and saprophytic growth starts subsequently from the dead host. Finally, sporulation of the fungus takes place a few days later, depending on environmental conditions [10].

More specifically, the hypocrealean entomopathogenic fungi *Beauveria bassiana* and *Metarhizium anisopliae* can reduce the lifespan of mosquitoes under laboratory and field conditions [11], and they are also equally effective in killing insecticide-resistant mosquitoes [12–14]. Besides the lethal effects, fungal infections reduce rodent *Plasmodium* sporozoite levels [15], female fecundity and feeding propensity [16, 17]. Most likely, the latter is the result of entomopathogenic fungi that reduce the host-seeking behaviour of mosquitoes by lowering the response of olfactory receptor neurons exposed to odour cues, as demonstrated for *Anopheles stephensi*[18].

Entomopathogenic fungi kill mosquitoes relatively slowly compared to insecticides (1–2 weeks *vs* 1–2 days). This is potentially beneficial for controlling malaria, because the *Plasmodium* development time in the mosquito is about ten to 14 days [19]. Because of the delayed mortality, fungal pathogens specifically kill those mosquitoes that are old enough to transmit the parasite. Therefore, the selective pressure for survival in mosquitoes is reduced, and thus, the probability of developing resistance against fungal infections is much lower [20].

*Beauveria bassiana* is a cosmopolitan fungus from which more than thousand isolates have been collected from different locations and different points in time worldwide [21]. Phylogenetic analysis has revealed at least 18 different clades (S A Rehner, pers comm). However, to date it is not clear how much these fungal isolates vary in their ability to kill mosquitoes. The majority of mosquito control studies has focused on one isolate, namely IMI 391510 [12, 17, 22, 23] (except Blanford *et al.*[15]). It is important to study the natural variation of virulence against mosquitoes because this has an impact on the choices for selecting the optimal fungal agents for controlling malaria vectors and other mosquitoes and their diseases. The natural occurring variation in virulence can also be used to uncover the mechanisms that underpin it, which will allow the estimation of the potential for resistance development in the vector. For example, the whole genome of isolates with contrasting variation in virulence could be sequenced, and pairwise polymorphisms, deletions and Single Nucleotide Polymorphisms (SNPs) in structural and regulatory parts of genes could be analysed. Furthermore, transcriptome analysis of contrasting isolates could be performed, and linking the DNA-sequence variation to variation in gene expression could provide unique data on the fungal genes involved in insect infection and the molecular genetic mechanisms which influence virulence. This, in turn, will increase the potential for the development of entomopathogenic fungi as a biological control method against mosquitoes.

On the other hand, previous studies have shown that morphological and physiological characteristics of fungi are related to their virulence, such as hyphal growth rate, conidial viability, conidia production, conidia size, enzyme secretion among other factors [24–28]. For instance, fungal isolates with rapid germination and high hyphal growth rate could be desirable because such fungi could infect the host more rapidly [24]. Such characteristics could be used as criteria for isolate selection in addition to their virulence towards mosquitoes.

Therefore, in the current study, the virulence of 29 isolates of *B. bassiana* was evaluated on the malaria mosquito *Anopheles coluzzii* (formerly: *Anopheles gambiae sensu stricto*). Phenotypic characteristics of the fungal isolates such as sporulation, spore size and growth rate were measured and their correlation with virulence analyzed.

## Methods

### Mosquito rearing

*Anopheles coluzzii* mosquitoes used in the experiments originated from Suakoko, Liberia (courtesy of the late Prof M Coluzzi). Larvae were reared in plastic trays of 10 × 25 × 8 cm, filled with 1 l of tap water at densities of approximately 0.3 larvae/cm^2^. Larvae were fed on Tetramin® fish food (Tetra A G, Melle, Germany) daily, using 0.1 mg/larva for first instar larvae and 0.3 mg/larva for the other three larval stages. Pupae were collected daily and transferred to holding cages of 30 × 30 × 30 cm in which adults were maintained in climate controlled rooms (27 ± 1°C, 80 ± 10% RH and a 12 hr L:D) and fed *ad libitum* on a 6% glucose solution.

### Production of *Beauveria bassiana*

All isolates of *B. bassiana* used in this study were obtained from the USDA-ARS Collection of Entomopathogenic Fungal Cultures (ARSEF) (Table 1) except for isolate IMI 391510, which was kindly provided by the Bioprocess Engineering Department (Wageningen University, The Netherlands). Isolates were selected randomly from the ARSEF collection taking into account a wide geographical distribution as well as a broad array of hosts, including isolates that were found in soil. Initially the fungi were grown on Sabouraud Dextrose Agar with 1% yeast extract (SDAY) for 14 days at 27°C and spores were harvested with a 0.05% Tween 80 solution to make a spore suspension and kept at -80°C until used.

**Table 1:** **Details of the tested** ***Beauveria bassiana*** **isolates against** ***Anopheles coluzzii*** **, including information from which host insect it was isolated, geographic origins and morphological characteristics**

Isolate ^1^	Host insect	Geographic origins	Size (μm) (±SEM)	Growth (mm/day) (±SEM)	Sporulation (x 10 ^6^ conidia/cm ^2^ ) (±SEM)
**ARSEF 220**		Commonwealth of Independent States	2.57 (±0.02)	2.22 (±0.01)	3.42 (±1.79)
**ARSEF 502**	Lepidoptera; Pyralidae; *Ostrinia nubilalis*	China	2.75 (±0.03)	2.38 (±0.01)	9.75 (±0.32)
**ARSEF 714**	Homoptera; Delphacidae; *Nilaparvata lugens*	China: Wuhan, Hupei	2.34 (±0.00)	2.54 (±0.07)	14.69 (±2.00)
**ARSEF 721**	Coleoptera; Chrysomelidae; *Diabrotica sp.*	Colombia: Cali, Valle del Cauca	2.45 (±0.03)	2.14 (±0.02)	4.75 (±1.63)
**ARSEF 1149**	Lepidoptera; Noctuidae; *Helicoverpa armigera*	Spain: Córdoba	2.47 (±0.02)	2.36 (±0.05)	16.52 (±1.07)
**ARSEF 1514**	Diptera; Muscidae; *Musca autumnalis*	France: Le Trait, Seine-Maritime	2.52 (±0.03)	1.85 (±0.03)	1.51 (±0.36)
**ARSEF 1520**	Hemiptera; Miridae; *Lygus sp.*	France: Prades, Pyrénées-Orientales	2.45 (±0.01)	2.41 (±0.02)	6.83 (±0.36)
**ARSEF 1816**	Coleoptera; Curculionidae; *Sitona discoideus*	Morocco: Hauteban	2.49 (±0.02)	2.45 (±0.01)	31.72 (±9.84)
**ARSEF 2075**	Coleoptera; Chrysomelidae; *Ceratoma arcuata*	Brazil: Ribeira do Pombal, Bahia	2.55 (±0.01)	2.45 (±0.03)	9.37 (±2.12)
**ARSEF 2427**	Homoptera; Delphacidae; *Nilaparvata lugens*	Indonesia: Cikampek, Java Barat, Java	2.40 (±0.01)	2.60 (±0.02)	24.64 (±4.28)
**ARSEF 2571**	Lepidoptera; Lymantriidae; *Lymantria dispar*	USA: Delaware	2.39 (±0.02)	2.23 (±0.08)	13.46 (±1.93)
**ARSEF 2597**	Lepidoptera; Hyblaeidae; *Hyblaea puer*	India	2.37 (±0.01)	2.26 (±0.01)	3.02 (±0.31)
**ARSEF 2861**	Homoptera; Aphididae; *Diuraphis noxia*	USA: Parma, Idaho	2.38 (±0.01)	2.19 (±0.05)	4.16 (±1.09)
**ARSEF 4135**	Coleoptera; Scarabaeidae; *Adoryphorus coulonii*	Australia: Newtown, Tasmania	2.47 (±0.01)	2.30 (±0.05)	1.97 (±0.62)
**ARSEF 4305**	Soil	Australia: Epping Forest, Tasmania	2.43 (±0.02)	2.46 (±0.09)	14.74 (±1.78)
**ARSEF 4396**	Soil	Japan: Sapporo, Hokkaido	2.37 (±0.02)	2.71 (±0.02)	23.11 (±0.54)
**ARSEF 4672**	Lepidoptera; Hepialidae; *Oncopera intricata*	Australia: Plenty, Tasmania	2.23 (±0.01)	2.08 (±0.02)	1.08 (±0.08)
**ARSEF 5078**	Lepidoptera; Pyralidae; *Galleria mellonella*	USA: Grayland, Washington	2.41 (±0.03)	2.34 (±0.07)	2.83 (±0.44)
**ARSEF 5641**	Orthoptera; Acrididae; *Schistocerca gregaria*	Ethiopia: Eritrea, Shelsela	2.20 (±0.01)	2.27 (±0.03)	3.03 (±0.96)
**ARSEF 5642**	Orthoptera; Acrididae; *Schistocerca gregaria*	Ethiopia: Eritrea, Shelsela	2.20 (±0.02)	2.16 (±0.03)	5.08 (±0.68)
**ARSEF 5769**	Homoptera; Adelgidae; *Adelges tsugae*	USA: Lovingston, Virginia	2.23 (±0.01)	1.79 (±0.05)	24.82 (±5.89)
**ARSEF 6686**	Coleoptera; Scarabaeidae	Ethiopia: Tikur Inchini, Western Shoa	2.46 (±0.01)	2.04 (±0.03)	4.83 (±1.26)
**ARSEF 6907**	Isoptera; Rhinotermitidae; *Coptotermes formosanus*	USA: Lake Charles, Louisiana	1.95 (±0.01)	2.45 (±0.05)	10.29 (±1.51)
**ARSEF 8028**	Hemiptera: Anthocoridae, *Anthocoris nemorum*	Denmark: Bakkegården, Copenhagen, Tåstrup, Zealand	2.26 (±0.01)	1.77 (±0.03)	0.74 (±0.08)
**ARSEF 8034**	Lepidoptera; Pyralidae; *Galleria mellonella*	Denmark: Bakkegården, Copenhagen, Tåstrup, Zealand	2.62 (±0.05)	1.77 (±0.06)	0.97 (±0.28)
**ARSEF 8414**	Coleoptera; Cerambycidae, *Anoplophora glabripennis*	China: Wuhe, Anhui	2.28 (±0.02)	2.27 (±0.10)	8.78 (±0.82)
**ARSEF 8854**	Coleoptera; Scarabaeidae. *Rhopaea magnicornis*	Australia: Condong, New South Wales	2.48 (±0.03)	2.86 (±0.07)	13.44 (±3.37)
**ARSEF 9595**	Hymenoptera; Apoidea	China: Guizhou Province	2.55 (±0.02)	1.65 (±0.10)	0.35 (±0.07)
**IMI 391510**	Coleoptera: Chrysomelidae	USA	2.34 (±0.01)	2.37 (±0.07)	6.82 (±0.84)

^1^ARSEF: USDA-ARS Collection of Entomopathogenic Fungal Cultures, USA. IMI: CABI Bioscience, UK.

To have sufficient spores for the experiments, all isolates were grown on solid-state fermenters, with a final yield of around 1–3 g of air-dry spores per isolate. In brief, 50 g hemp was weighed and 200 ml of distilled water was added, mixed and sterilized for 30 min at 121°C. Then 10 g of peptone and yeast extract were added and sterilized again. Once this mixture was cold, 200 ml of a pre-autoclaved solution of glucose (36.5 w/w%) was added. This mixture was inoculated with 1 ml of spore suspension (1 × 10^8^ spores/ml). It was incubated overnight at room temperature on a roller-bank. Then, the hemp was transferred into a 0.2 l glass tube (4.6 cm diameter; 30 cm height) and placed in a climate chamber set at 25°C. After 21 days, spores were dried for four days by blowing dry air through the glass tube that contained the mixture. Spores were then harvested with sieves of various sizes (125 μm-1 mm), and stored in 50 ml tubes. The spores were kept at 4°C in the dark prior to use.

### Bioassays

The virulence of 29 isolates of *B. bassiana* was checked by doing seven bioassays. Between three and six isolates were simultaneously compared in each bioassay. The seventh bioassay included four randomly chosen isolates to check for temporal experimental variation. In every bioassay, fungal spores of each isolate were suspended in Shellsol T® oil (Shell, The Netherlands) and standardized to 1 x 10^9^ spores/ml by adjusting spore concentration after counting the suspension with a Bürker-Türk counting chamber. Spore viability was checked on SDAY plates after 18–20 hrs at 27°C, and spores with a detectable germ tube were considered viable. Of the standardized spore solution, 0.9 ml was applied to an A4-sized proofing paper one day before exposure. Coated papers were left to dry in a fume hood [29]. These papers were then placed in PVC tubes (15 cm height and 8 cm diameter) and sealed with cling film on both ends. For each isolate tested, three PVC tubes (replicates) were prepared in this way. As a control, papers were coated with Shellsol T® oil only. Thirty *An. coluzzii* female mosquitoes (3–5 days old) were transferred with an aspirator to each PVC tube with the coated papers and exposed for three hours. After this, mosquitoes were transferred to plastic buckets (25 cm height and 20 cm diameter), which were sealed with a nylon sock. All buckets were kept in a climate controlled chamber (27 ± 1°C, 80 ± 10% RH, 12 h L:D), and daily mortality was checked for 14 days. Mosquitoes were fed *ad libitum* with a 6% glucose solution on a cotton plug. Fungal infection of dead mosquitoes was checked by dipping them for 5 sec in 70% ethanol, incubating them on moist filter paper in sealed petri dishes at 25°C for five to seven days, and inspecting them for visible fungal growth.

### Phenotypic characterization of fungal isolates

To determine spore size, isolates were grown on SDAY petri dishes for 14 days and spores were harvested with a 0.05% Tween 80 solution to make a spore suspension. A 1:50 dilution was made and size was measured for four replicates per fungal isolate with a Coulter Counter Z2 (Beckman Coulter). Linear growth rate was measured using ‘race tubes’ [30]. The tubes were filled with 23 ml of SDAY medium. Three tubes were inoculated for each isolate and growth was measured every week for three months. To determine sporulation, spores were harvested from a 14-day-old culture on SDAY with 0.05% Tween 80 solution and counted on a haemocytometer chamber using an optical microscope (400X). Two areas of 1 mm^2^ were counted for each plate, and three plates in total were counted for each isolate.

### Statistical analysis

Mosquito survival was analysed using Kaplan-Meier survival analysis in SPSS (v.19) with significant differences between different isolates estimated using a Log Rank Test. Differences between the control and the infected mosquitoes were examined using a Cox Regression analysis in SPSS. Hazard ratios (HR; the daily chance of death) in comparison with the isolate IMI391510 were calculated. One-way ANOVA was conducted in R (2.12.2) to detect differences in conidia size, sporulation and linear growth rate, with the sporulation data being Log transformed before analysis. A General Lineal Model was used to analyse the correlation between phenotypic characteristics of isolates and their virulence on mosquitoes using R (2.12.2). To further check for any correlation among the phenotypic characteristics themselves, a Pearson’s Correlation analysis was done. Mosquito survival exposed to the same fungal isolates at different time points were analysed using Kaplan-Meier survival analysis in SPSS (v.19) and significant differences were estimated using a Log Rank Test.

## Results

### Bioassays on *Anopheles coluzzii*

**Figure 1:**
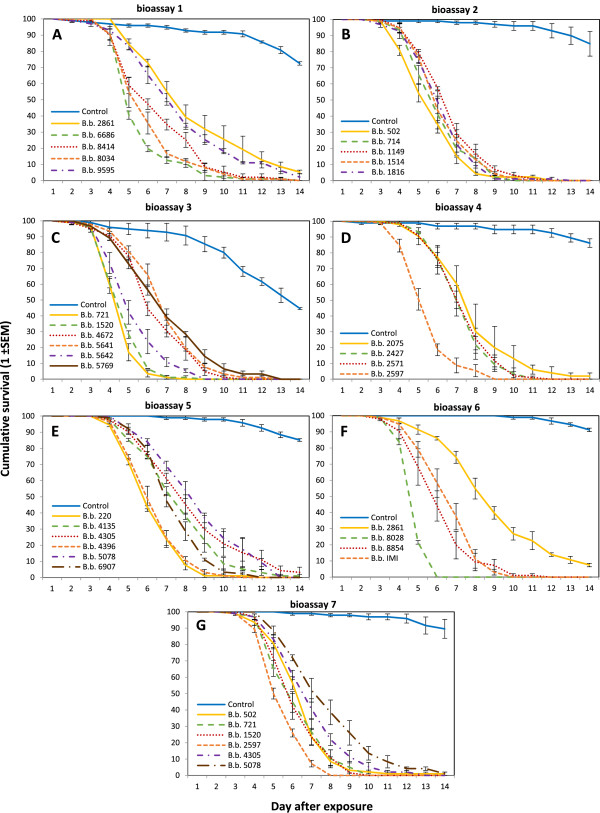
**Cumulative daily proportional survival of** ***Anopheles coluzzii*** **exposed to spores of different** ***Beauveria bassiana*** **isolates.** Control (dark blue) were exposed to only Shellsol T® oil. Exposures were carried out in seven bioassays **(A-G)** and 29 isolates were tested in total. Data show means ± SEM from three replicates of 30 female mosquitoes.

All *B. bassiana* isolates tested were pathogenic to *An. coluzzii* with mortalities of at least 92.5% by day 14 (Figure 1). Survival curves for all mosquitoes infected with the fungus were significantly different from the respective controls for each of the seven bioassays (Figure 1). Furthermore, 74-100% of the dead mosquitoes that were exposed to fungus-coated paper showed evidence of fungal infection in the form of sporulation after five to seven days. The viability of fungal conidia of each fungal isolate was always higher than 70% at the start of the experiment. Although all bioassays were conducted in the same manner, not all controls behaved similarly in all bioassays. For unknown reasons, control mosquitoes from bioassay 1 and 3 had a significantly higher mortality compared to the controls from the other five bioassays (F = 96.09, d.f. = 6, p < 0.001; Figure 1A, 1C). Therefore, these two bioassays were not considered for further analysis.

**Figure 2:**
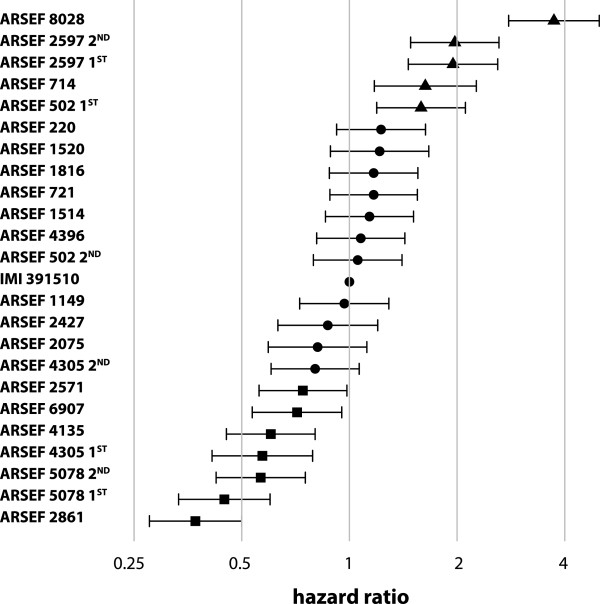
**Hazard ratios for fungal infection using isolate IMI391510 as a baseline.** Dot symbols represent the isolates that were not significantly different from the reference isolate IMI391510. Triangle/square symbols show isolates that were more/less virulent than the reference isolate. Whiskers represent the 95% CIs. 1^st^ or 2^nd^ indicates the result of the first or second biological replicate of an isolate.

Hazard ratios (HR) were estimated relative to the mortality pattern of the reference isolate, IMI391510. This isolate was tested in detail during earlier laboratory and field trials [11, 23], and thus constituted a relevant reference point. Virulence was classified in three groups: (1) isolates for which the HR was significantly higher than the HR of the reference isolate (these were termed ‘highly lethal’ (HL)); (2) isolates that were not significantly different compared the reference isolate (these were termed ‘lethal’ (L)); and, (3) fungal isolates that had significantly lower HR than the reference isolate (these were termed ‘slightly lethal’ (SL)). The majority of the 29 isolates was classified as lethal, although at least three were HL isolates and five were SL isolates (Figure 2). Virulence varied widely, as depicted by the extremes: isolate 8028 was on average 3.7 times more virulent and isolate 2861 was on average 2.7 times less virulent than the reference isolate IMI391510. All of these isolates (8028, IMI391510 and 2861) were tested within the same bioassay (Figure 1F) removing any possibility of temporal bias.

Isolates 502, 2597, 4305, and 5078 were randomly selected for the seventh bioassay to assess the presence of temporal variation in virulence. The two replicates of 2597 and 5078 were not significantly different from each other between the two time points and therefore were classified in the same virulence group (HL and SL, respectively). However, isolates 502 and 4305 showed significantly different HR between the temporal replicates (*x*^*2*^ = 10.33, d.f. = 1, p = 0.001; *x*^*2*^ = 6.56, d.f. = 1, p = 0.010), and thus, their virulence status changed. Isolate 502 was considered HL in the first trial, and then L in the second trial. The opposite effect was observed for isolate 4305.

### Phenotypic characterization of fungal isolates

Conidia of the 29 isolates of *B. bassiana* had significant differences in size, ranging from 1.95 to 2.75 μm (F = 67.33, d.f. = 28, p < 0.0001, Table 1). Conidia of isolates 502 and 8034 were the largest in size, whereas conidia of isolate 6907 were the smallest. Sporulation ranged from 3.5 x10^5^ to 3.17 x10^7^ spores/cm^2^, and growth rate from 1.77 to 2.71 mm/day, with isolates differing significantly for both traits (F = 10.08, d.f. = 28, p < 0.0001, and F = 11.48, d.f. = 28, p < 0.0001, respectively). The isolate that grew fastest was 4396 and the isolate that produced the most conidia was 1816. Nevertheless, none of these phenotypic characteristics of *B. bassiana* that were studied were correlated to the ability to kill malaria mosquitoes (i.e. virulence): sporulation (F = 1.17, d.f. = 28, p = 0.791), growth rate (F = 25.47, d.f. = 28, p = 0.195) and spore size (F = 22.74, d.f. = 28, p = 0.169). Additionally, a Pearson’s Correlation analysis was performed to further check for correlation among the phenotypic characteristics themselves, but none was found, except for a positive correlation between grow rate and sporulation (r = 0.704, p < 0.001).

## Discussion

Under laboratory conditions, all 29 tested isolates of *B. bassiana* killed *An. coluzzii* mosquitoes, although the rate at which this happened differed significantly among the isolates. The daily risk of death for mosquitoes when exposed to the most virulent isolate was around ten times higher than when exposed to the least virulent isolate. This observation is a substantial addition to a previous smaller scale study which tested six isolates of *B. bassiana* and two isolates of *Metarhizium anisopliae* against *Anopheles stephensi* mosquitoes [15]. In that study, of two isolates of *M. anisopliae* tested, only one was pathogenic to mosquitoes. Similarly, from six *B. bassiana* isolates, one was not pathogenic whereas the others had different rates of virulence.

Much progress has been made regarding the feasibility of using entomopathogenic fungi as a biological control agent for malaria mosquitoes. However, researchers have thus far overlooked the potential of selecting the most suitable isolate from the existing natural variation in fungal characteristics. From the study by Blanford *et al.*[15] onwards, isolate IMI391510 was selected in further experiments. This was based on practical considerations and not necessarily because it was the most virulent towards mosquitoes. For instance, this isolate was already evaluated and received regulatory approval for the control of other insects under field conditions. Follow-up studies mostly focused on isolate IMI391510 only or other isolates of *M. anisopliae*. These studies managed to standardize and improve their application under laboratory conditions [29], test it under field conditions [31], on insecticide-resistance mosquitoes [12–14], and on different substrates such as cloth, netting, wood, clay tiles, and mud walls [11, 17, 32]. In the present study, it has been shown the potential of selecting the most virulent isolate since this can result in an increased effectiveness of up to ten-fold. Such selection should be dependent on the ecological situation at the location of intended use, as using a highly virulent isolate may potentially lead to an increased selection pressure that can result in resistance in mosquitoes [3]. Additionally, genetic diversity of natural populations of mosquitoes have to be taken into consideration, since some genotypes could be more susceptible to fungal infection than others, and this could lead to an increased selective pressure as well [33]. Moreover, it has been shown that there is a dose-dependent effect of spore concentration [17, 29], so when comparing a highly lethal to a lethal isolate, the highly lethal isolate could have a similar effect using a lower concentration. Thus, the amount of spores can potentially be reduced, which would lower the application costs. Nevertheless, other factors need to be further considered since virulence is not the only variable that is important when using a fungus as biological control agent. Viability and persistence for example, can be limitations in tropical (malaria-endemic) climates.

It was hypothesized that phenotypic characteristics of *B. bassiana* potentially correlate with virulence in mosquitoes as previous studies have shown that morphological and physiological traits of *B. bassiana* are related to virulence in other insects [24, 25, 28]. For example, fungal isolates with rapid germination and high hyphal growth rate may be advantageous to use as a biological control agent, because such fungi could infect the host more rapidly [24]. Zhang *et al.*[28] found a positive correlation between growth rate, sporulation and germination rate of *B. bassiana* with virulence of red turpentine beetle larvae. Nevertheless, in the current study no relationship could be demonstrated between morphological characteristics and the virulence of *B. bassiana* isolates to *An. coluzzii*. This is consistent with earlier work of Talaei-Hassanloui *et al.*[26] who did not find any correlation between radial growth rate, spore size, germination or pigmentation and virulence on second instar larvae of *Leptinotarsa decemlineata* and *Plutella xylostella*. Ideally, one would like to be able to predict virulence of a fungus from its phenotypic characteristics without conducting bioassays that require many live insects and relatively long experimental duration before results are known. However, no such trend was observed in this study. Therefore, bio-assays remain the method of choice to unravel fungal virulence, at least for this fungal-insect system and with this selection of isolates.

The results suggest that other molecular and physiological mechanisms, such as the excretion of chitinases and the ability to avoid and/or counter the insect immune response, could be related to the variation in fungal virulence. Indeed, the wide variation observed in virulence offers a unique opportunity to understand the genetic, molecular and physiological mechanisms that underpin this variation. In *B. bassiana* several proteins have been characterized as relevant for virulence. For instance, proteins of the P450 family have been identified as important for cuticle degradation [34, 35], and Bbslt2, a novel mitogen-activated protein (MAP) kinase, was identified to have a crucial role in regulating fungal development, growth and pathogenicity [36]. As well as Bbgas1, a gene encoding a putative transferase (Glycosylphosphatidylinositol-Anchored β-1,3-Glucanosyltransferase) is involved in conidial thermotolerance and virulence [37]. Many more genes have also been linked to virulence, which hint that virulence is a complex process regulated by several pathways.

## Conclusions

The results demonstrate that there is much natural variation in virulence of fungal isolates of *B. bassiana* that can be exploited by choosing the most suitable isolate for controlling malaria mosquitoes. This notion makes the use of entomopathogenic fungi an even more viable option, especially if used as part of an integrated vector management strategy. In addition, the natural variation observed in virulence offers the possibility to focus on the genetic mechanisms that determine this variation and would help in understanding the fungal mechanisms of *B. bassiana* towards malaria mosquitoes, and provide evidence that this could be an evolution-proof biological control agent. This is where the current research effort is focusing.
